# Presentations of patients of poisoning and predictors of poisoning-related fatality: Findings from a hospital-based prospective study

**DOI:** 10.1186/1471-2458-8-7

**Published:** 2008-01-08

**Authors:** Hsin-Ling Lee, Hung-Jung Lin, Steve Ting-Yuan Yeh, Chih-Hsien Chi, How-Ran Guo

**Affiliations:** 1 Graduate Institute of Clinical Medicine, College of Medicine, National Cheng Kung University, 138 Sheng-Li Road, Tainan City 704, Taiwan; 2 Department of Occupational and Environmental Medicine, College of Medicine, National Cheng Kung University, 138 Sheng-Li Road, Tainan City 704, Taiwan; 3 Department of Occupational and Environmental Medicine, National Cheng Kung University Hospital, 138 Sheng-Li Road, Tainan City 704, Taiwan; 4 Graduate Institute of Injury Prevention and Control, Taipei Medical University, 252 Wu-Hsing Street, Taipei City 110, Taiwan; 5 Department of Emergency Medicine, Chi-Mei Medical Center, 901 Chung-Hwa Road, Yongkang City, Tainan County 710, Taiwan; 6 Department of Emergency Medicine, National Cheng Kung University Hospital, 138 Sheng-Li Road, Tainan City 704, Taiwan; 7 Department of Environmental and Occupational Health, College of Medicine, National Cheng Kung University, 138 Sheng-Li Road, Tainan City 704, Taiwan

## Abstract

**Background:**

Poisoning is a significant public health problem worldwide and is one of the most common reasons for visiting emergency departments (EDs), but factors that help to predict overall poisoning-related fatality have rarely been elucidated. Using 1512 subjects from a hospital-based study, we sought to describe the demographic and clinical characteristics of poisoning patients and to identify predictors for poisoning-related fatality.

**Methods:**

Between January 2001 and December 2002 we prospectively recruited poisoning patients through the EDs of two medical centers in southwest Taiwan. Interviews were conducted with patients within 24 hours after admission to collect relevant information. We made comparisons between survival and fatality cases, and used logistic regressions to identify predictors of fatality.

**Results:**

A total of 1512 poisoning cases were recorded at the EDs during the study period, corresponding to an average of 4.2 poisonings per 1000 ED visits. These cases involved 828 women and 684 men with a mean age of 38.8 years, although most patients were between 19 and 50 years old (66.8%), and 29.4% were 19 to 30 years. Drugs were the dominant poisoning agents involved (49.9%), followed by pesticides (14.5%). Of the 1512 patients, 63 fatalities (4.2%) occurred. Paraquat exposure was associated with an extremely high fatality rate (72.1%). The significant predictors for fatality included age over 61 years, insufficient respiration, shock status, abnormal heart rate, abnormal body temperature, suicidal intent and paraquat exposure.

**Conclusion:**

In addition to well-recognized risk factors for fatality in clinical settings, such as old age and abnormal vital signs, we found that suicidal intent and ingestion of paraquat were significant predictors of poisoning-related fatality. Identification of these predictors may help risk stratification and the development of preventive interventions.

## Background

Poisoning is a public health problem worldwide [1-9] and is one of the most common reasons for attendance at hospital emergency departments (EDs) [6,10-12]. Although the incidence of poisoning is difficult to estimate accurately, the wide availability and accessibility of chemicals and their extensive use in a variety of applications including medicine, agriculture and industry has increased the risk of poisoning [1,2,13].

Knowledge of the epidemiology of poisoning and its changes is important to both emergency physicians and public health practitioners, but the pattern of poisoning varies from country to country and over time, sometimes rapidly [1,2,13-16]. Therefore, regional epidemiological data on poisoning are very helpful in planning rational use of resources for the prevention and management of poisoning and in targeting research. Various epidemiological studies concerning poisoning have been undertaken including single hospital-based, multi-center-based and poison center-based investigations [1,5,6,17,18].

Poisoning is common in Taiwan but epidemiological studies and surveillance data are limited [2,19,20]. According to the Poison Control Center (PCC) the annual incidence of poison exposure in Taiwan ranges from 0.16 to 0.22 exposures per 1000 population [2]. The PCC receives calls on poisoning and poison exposure from all over the country, but for various reasons not all poisoning cases are reported to the PCC [21,22]; this is considered a major factor contributing to differences in estimates between those reported by the PCC and other epidemiologic studies. There is little current systematic information on the causes, circumstances and clinical course of poisoning in the southwest region of Taiwan.

In developed countries, poisoning exposure has mostly been associated with medicines, alcohol and household chemicals [1,4,6], whereas in developing countries the common causal agents are agrochemicals, including pesticides [13,17,23-25]. Pesticide exposure and pesticide-related morbidity and fatality have been, and continue to be, a major concern in acute poisonings in Taiwan. A number of studies concerning identification of prognostic factors and assessment of severity have been reported [24,26-31], but most have focused on prognostic factors for a single type of pesticide, and have evaluated the impact using clinical scoring systems such as the Simplified Acute Physiology Score (SAPS) II and Acute Physiology and Chronic Health Evaluation (APACHE) II and III [26,30-33]. Factors that help to predict overall poisoning-related fatality formed the basis of the present study, as these have rarely been elucidated.

We speculated that factors generally associated with unfavorable prognosis in clinical settings, such as unstable vital signs (including decreased consciousness, shock status, respiratory distress, abnormal heart rate and abnormal body temperature), history of major disease, and old age are predictors of fatality in cases of poisoning. However, other patient characteristics may also be associated with poisoning-related fatality. The objectives of this study were to describe the demographic and clinical characteristics in poisoning cases, and to compare instances of poisoning-related fatality and survival to identify and evaluate predictors of fatality.

## Methods

### Study design, setting, and selection of the study population

We conducted a prospective study involving recruited consecutive cases of acute and chronic poisoning exposure in children and adults who presented between January 1, 2001 and December 31, 2002 to the EDs of the only two medical centers in the Tainan area. This area is in the southwest region of Taiwan, includes Tainan City and the Tainan County, and had a population of about 1,850,000 during the study period. The study proposal was reviewed and approved by the Research Grant Review Committee of National Science Council, Taiwan, R.O.C., and the Research Grant Review Committee of the National Cheng Kung University.

### Data collection and processing

All consecutive cases of poisoning exposure, including cases with adverse drug effects of prescribed medication were enrolled within 24 hours of arrival at the ED, but cases diagnosed as food poisoning were excluded. We also enrolled patients who died on arrival. Poisoning was defined as exposure to drugs or any environmental substance resulting in an ED visit, even if the patient was considered symptom-free by the ED staff. Acute exposure was defined as a single exposure, continuous exposure lasting less than 8 hours, or repeated exposures over a period no longer than one week [2]. Chronic exposure was defined as continuous exposure lasting for 8 hours or more, or repeated exposures over a period longer than one week [1,2]. A poisoning report form was developed for data collection, and periodically evaluated and refined on the basis of preliminary data. The information collected included demographic data; exposure agents, time, types, routes and reasons; history of suicide attempts, psychiatric diseases, substance abuse or major systemic disease; presenting symptoms and signs; details of psychiatric consultation; methods of management; clinical observations; ED and final outcomes; and laboratory data. Poisoning report forms were completed for all enrolled patients except those diagnosed with food poisoning. Report form data were initially obtained through direct interview of patients or patient caregivers by the ED staff and medical assistants. The reports were completed by trained assistants who followed the patients during hospitalization. Those who were discharged with stable condition were routinely followed by the psychiatric or toxicological outpatient clinic for at least once, and therefore we could obtain information on each patient's condition after discharge. For those who were discharged with unstable condition, were transferred to other hospitals, or could not return to the outpatient clinics, follow-up phone calls were made to the patients or their friends or relatives to obtain information.

According the practice of the two participating EDs, all poisoning cases receive psychiatric consultation or social worker consultation, and therefore, the suicide intents were assessed by professional staff. For those who were unable to be assessed in the ED, the consultation would be performed after their conditions were stabilized. On most of the critically ill and die-on-arrival patients, we managed to obtain information from their friends, relatives, emergency paramedical technicians, and policemen.

### Outcome measures

All enrolled patients were tracked throughout the hospitalization period and during follow-up to document final outcomes. These were categorized as survival or poisoning-related fatality, the latter being defined as death during hospitalization or death after discharge that could be attributable to the poisoning episode.

### Primary data analysis

The potential poisoning-related fatality predictors evaluated in this study included demographic characteristics (age and gender), exposure types (acute or chronic), nature of the exposure agents, the number of exposure agents (multiple or single), suicidal intent as the cause of poisoning, and concomitant use of alcohol. Vital signs (initially checked on the patient's arrival at the ED) including consciousness level, body temperature (BT), respiration pattern and rate, blood pressure and heart rate were also assessed. Past medical history of attempted suicide, psychiatric disease (e.g., schizophrenia, major depression, sleeping disorder), and other major or chronic diseases (cancers and diseases for which long-term medication is needed) was obtained from the patient or medical records. Laboratory data also recorded for reference included blood-cell counts, liver and renal function, blood gas analyses and x-rays.

We analyzed the data using SPSS statistical software (11.5.0; SPSS Inc., Chicago, IL). The exploratory data analyses checked the distribution of values and presented the results as the mean and standard deviation (SD) for numerical data, and a proportion (%) for nominal data. Differences between survivors and fatalities were tested using the chi-square test or Fisher's exact test for categorical variables, and *t *test for continuous variables. A *p *value < 0.05 was considered statistically significant, and all the statistical tests were two-tailed. We calculated the odds ratios (ORs) to assess the relationship between potential predictors and outcomes. Multiple logistic regression analyses were applied to adjust for confounders, to obtain adjusted estimates of OR and the 95% confidence interval (CI) for the independent effects of the potential predictors, and to identify significant independent predictors of outcomes.

## Results

### Characteristics of the poisoning cases

**Figure 1:**
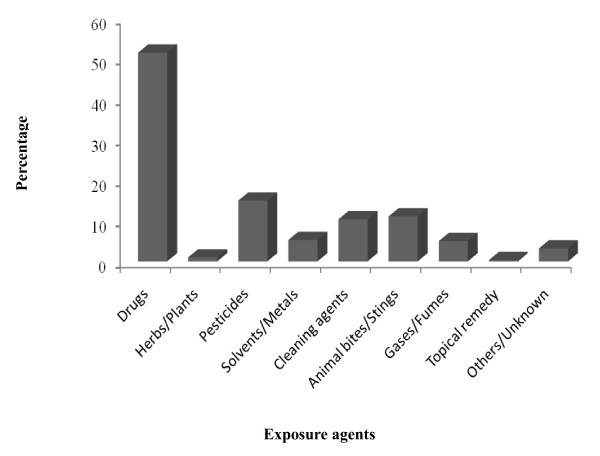
Categories of exposure agent.

From January 1, 2001 to December 31, 2002, a total of 1512 individuals presented with poisoning to the ED of the two medical centers, corresponding to an average of 4.2 per 1000 ED visits. The estimated incidence of poisoning events was around 0.41 poisoning exposures per 1000 population served in the Tainan area annually. Acute poisoning accounted for 98.0% of the enrolled poisoning cases, and involved more women (828) than men (684). Most cases involved people between 19 and 50 years of age (66.8%), and particularly those 19–30 years (29.4%). The mean age was 38.8 years (SD = 18.8, median = 35). Of 1548 poisoning agents implicated during the study period, drugs dominated in all age groups (49.9%), followed by pesticides (14.5%) (Figure 1). Overall, 66.1% of the poisoning exposures involved suicidal intent. Concomitant use of alcohol was recorded for 62.4% of the enrolled patients, and 17.3% of them used alcohol in the episode (Table 1).

**Table 1:** General characteristics of the 1512 poisoning patients involved in the study.

**Parameters**	**Fatalities (N = 63)**	**Survivors (N = 1449)**	**Subtotal (%)**	**OR (95%CI)**	***p *value**
**Age group (years)**			(N = 1493)		< 0.01
Age ≥ 61	27	218	245 (16.4)	4.3 (2.6–7.2)	
Age < 61	35	1213	1248 (83.6)	1	
**Gender**			(N = 1512)		< 0.01
Male	42	642	684 (45.2)	2.5 (1.5–4.3)	
Female	21	807	828 (54.8)	1	
**Type of exposure**			(N = 1512)		0.52
Acute	62	1420	1482 (98.0)	2.1 (0.3–16.4)	
Chronic	1	11	12 (0.8)	1	
Undetermined	0	18	18 (1.2)	-	
**Number of Agents**			(N = 1456)		0.07
Multiple agents	4	204	208 (14.4)	0.4 (0.1–1.1)	
Single agent	59	1189	1248 (85.6)	1	
History of chronic or major diseases*			(N = 1512)		< 0.01
Yes	25	287	312 (20.6)	2.7 (1.6–4.5)	
No	38	1162	1200 (79.4)	1	
**History of psychiatric diseases**			(N = 1512)		0.28
Yes	16	462	478 (31.6)	0.7 (0.4–1.3)	
No	47	987	1034 (68.4)	1	
**Suicidal intent**			(N = 1512)		< 0.01
Yes	58	941	999 (66.1)	6.3 (2.5–15.7)	
No	5	508	513 (33.9)	1	
**History of suicidal attempts**			(N = 1082)		0.18
Yes	5	247	252 (23.3)	0.5 (0.2–1.4)	
No	31	799	830 (76.7)	1	
**Concomitant use of alcohol**			(N = 943)		0.08
Yes	2	161	163 (17.3)	0.3 (0.1–1.3)	
No	31	749	780 (82.7)	1	

Notes: OR = odds ratio, SD = standard deviation, CI = confidence interval.

*Chronic or major diseases included cancers and diseases for which long-term medication is needed, but did not include psychiatric diseases.

### Clinical status of the poisoning cases

Poisoning patients presenting to the medical center included individuals with impaired consciousness (14.0%; Glasgow Coma Scale (GCS) less than 10), abnormal body temperature (24.6%) including hyperthermia (BT ≥ 37.5°C) and hypothermia (BT < 36.0°C), abnormal heart rate (12.8%) including tachycardia (heart rate > 120 beats/min) and bradycardia (heart rate < 60 beats/min), shock status (3.0%; defined as systolic blood pressure < 90 mmHg), and insufficient respiration (6.0%; defined as respiratory distress necessitating intubation or involving a respiratory rate > 24 or < 10 breaths per minute) (Table 2).

**Table 2:** Clinical status of the 1512 poisoning patients on arrival at the emergency room.

**Parameters**	**Fatalities (N = 63)**	**Survivors (N = 1449)**	**Subtotal (%)**	**OR (95% CI)**	***p *value**
**Consciousness level**			(N = 1512)		< 0.01
GCS < 10	25	186	211 (14.0)	4.5 (2.6–7.6)	
GCS ≥ 10	38	1263	1301 (86.0)	1	
**Body temperature (BT)**			(N = 1475)		< 0.01
Hyperthermia (≥ 37.5°C) or hypothermia (< 36.0°C)	32	331	363 (24.6)	4.6 (2.6–7.9)	
BT 36.0–37.5°C	23	1089	1112 (75.4)	1	
**Heart rate (HR)**			(N = 1468)		< 0.01
Tachycardia (> 120 beats/min) or bradycardia (< 60 beats/min)	25	163	188 (12.8)	5.0 (3.0–8.5)	
HR 60 to 120 beats/min	38	1243	1281 (87.2)	1	
**Systolic blood pressure (SBP)**			(N = 1458)		< 0.01*
Hypotension (SBP < 90 mmHg)	14	30	44 (3.0)	13.6 (6.8–27.3)	
SBP ≥ 90 mmHg	47	1367	1414 (97.0)	1	
**Respiratory rate (RR)**			(N = 1461)		< 0.01*
Respiratory distress or necessity of intubation	27	60	87 (6.0)	17.7 (10.1–31.3)	
RR 10 to 24 per minute	34	1340	1374 (94.0)	1	

Notes: GCS = Glasgow Coma Scale, OR = odds ratio, CI = confidence interval, respiratory distress is defined as a respiratory rate of > 24 or <10 breaths per minute, or requiring intubation.

*Fisher's exact test.

### Characteristics of the fatality cases

There were 63 poisoning-related fatalities, including 9 died on arrival, 2 died at ED, and 52 died during hospitalization. Among the 63 poisoning-related fatalities (case fatality rate 4.2%) there was a predominance of men (66.7%), suicidal cases (92.1%), and older patients (53.9 ± 18.8 years for fatalities, 38.2 ± 18.6 for survivors; *p *< 0.01) (Table 1). Of the poisoning agents involved, pesticides caused the highest fatality rate (20.3% of 227 exposed). Paraquat, glyphosate, organophosphates, carbamates and blasticidin-S were the pesticides most frequently involved in fatal exposures and accounted for 73.0% of the fatalities; paraquat exposure caused the highest fatality rate (31 of 43 [72.1%] exposed) and was the major agent involved in fatalities (46.3%) (Table 3).

**Table 3:** Leading agents in the 63 poison-related fatalities involved in the study.

**Exposed agents**	**Fatalities^**† **^N = 63**	**Survivors N = 1449**	**Total N = 1512**	**OR (95% CI)**	***p *value**
**Hypnotics and sedatives**					
Yes/No	3^‡^/60	489/960	492/1020	0.10 (0.03 – 0.32)	< 0.01*
**Tricyclic antidepressants**					
Yes/No	1/62	12/1437	13/1499	1.9 (0.3 – 15.1)	0.43
**Digoxin**					
Yes/No	1/62	7/1442	8/1504	3.5 (0.4 – 28.6)	0.28
**Morphine**					
Yes/No	1/62	9/1440	10/1502	2.6 (0.3 – 20.7)	0.38
**Paraquat**					
Yes/No	31/32	12/1437	43/1469	116.0 (54.6 – 246.3)	< 0.01
**Glyphosate**					
Yes/No	4/59	37/1412	41/1471	2.6 (0.9 – 7.5)	0.09
**Organophosphates**					
Yes/No	7/56	68/1381	75/1437	2.5 (1.1 – 5.8)	0.02
**Carbamates**					
Yes/No	4/59	23/1426	63/1449	4.2 (1.4 – 12.5)	0.01
**Blasticidin-S (Fungicides)**					
Yes/No	2/61	1/1448	3/1509	47.5 (4.2 – 530.7)	< 0.01
**Potassium cyanide (KCN)**					
Yes/No	2/61	1/1448	3/1509	47.5 (4.2 – 530.7)	< 0.01
**Detergents**					
Yes/No	4/59	105/1344	109/1403	0.8 (0.3 – 2.3)	> 0.95
**Rodenticides**					
Yes/No	1/62	17/1432	18/1494	1.4 (0.2 – 10.4)	0.54
**Carbon monoxide (CO)**					
Yes/No	6/57	36/1413	42/1470	4.1 (1.7 – 10.2)	< 0.01

*Fisher's exact test was performed for analyses of all agents except for hypnotics and sedatives, for which the Pearson chi-square test was used.

^†^There were four fatalities involving exposure to two agents: paraquat and carbamates; organophosphates and carbamates; detergents and rodenticides; hypnotic and sedative drugs and CO.

^‡^One of three fatalities committed suicide by two methods – self-injuries (hanging and wrist-cutting) and ingestion of hypnotics and sedatives. This case died in the intensive care unit on the day of poisoning.

### Predictors of fatality

Univariate analyses identified the following significant predictors for poisoning-related fatalities: age ≥ 61 years (OR 4.3, 95% CI 2.6–7.2), male gender (OR 2.5, 95% CI 1.5–4.3), attempted suicide (OR 6.3, 95% CI 2.5–15.7), history of chronic or major disease (OR 2.7, 95% CI 1.6 – 4.5), abnormal vital signs (decreased GCS, abnormal body temperature, shock status, abnormal heart rate, and insufficient respiration), and exposure agents (paraquat, organophosphates, carbamates, blasticidine-S, KCN, CO, and hypnotics/sedatives) (Tables 1, 2 and 3).

We further evaluated predictors of poisoning-related fatality using multiple logistic regression analyses. This was done firstly in a "full model" by including in the univariate analyses all predictors with a *p*-value < 0.05, and then in a "final model," which included only the statistically significant predictors in the multivariate analyses (Table 4). As shock status correlated well with abnormal heart rate, we presented two final models, each including only one of these two parameters. Other significant independent predictors of poisoning-related fatality include paraquat exposure, insufficient respiration, abnormal body temperature, age ≥ 61 years, and suicidal intent. Exposure to hypnotics/sedatives was found to be associated with a significantly lower risk of fatality in both final models.

**Table 4:** Prognostic factors associated with poisoning fatality.

**Parameters**	**Full model OR (95% CI)**	Final Model 1* OR (95%CI)	Final Model 2* OR (95%CI)
Exposure to paraquat	116.0 (54.6–246.3)	192.3 (64.3–574.5)	194.4 (62.8–601.6)
Exposure to blasticidin-S	47.5 (4.2–530.7)	-	-
Exposure to KCN	47.5 (4.2–530.7)	-	-
Exposure to carbamates	4.2 (1.4–12.5)	-	-
Exposure to CO	4.1 (1.7–10.2)	-	-
Exposure to organophosphates	2.5 (1.1–5.8)	-	-
Exposure to hypnotics and sedatives	0.10 (0.03–0.32)	0.10 (0.01–0.82)	0.12 (0.02–0.91)
Insufficient respiration	17.7 (10.1–31.3)	7.6 (2.8–20.3)	7.6 (2.9–19.7)
Shock status	13.6 (6.8–27.3)	22.0 (6.2–77.9)	-
Abnormal heart rates	5.0 (3.0–8.5)	-	3.8 (1.4–10.5)
Abnormal body temperature	4.6 (2.6–7.9)	4.4 (1.8–10.7)	4.0 (1.7–9.5)
Consciousness level (GCS < 10)	4.5 (2.6–7.6)	-	-
Suicidal intent	6.3 (2.5 – 15.7)	7.5 (2.1 – 26.9)	9.9 (2.7 – 35.9)
Age ≥ 61 years	4.3 (2.6 – 7.2)	5.1 (2.0 – 12.6)	5.6 (2.3 – 13.8)
History of chronic or major diseases	2.7 (1.6 – 2.5)	-	-
Male gender	2.5 (1.5 – 4.3)	-	-

Notes: OR = odds ratio; CI = confidence interval; 'insufficient respiration' is defined as a respiratory rate of > 24 or < 10 breaths per minute, or requiring intubation; 'shock status' is defined as a systolic blood pressure <90 mmHg; 'abnormal heart rates' is defined as tachycardia (> 120 beats/min) or bradycardia (< 60 beats/min); 'abnormal body temperature' is defined as hyperthermia (body temperature ≥ 37.5°C) or hypothermia (body temperature < 36.0°C).

*The full models included all parameters with a *p *value < 0.05, but because 'abnormal heart rate' and 'shock status' were correlated, only one of the two parameters was included in each of the two models.

## Discussion

### Characteristics of the poisoning cases

The frequency of poisoning-related ED visits varies around the world, ranging from 0.76 to 24 per 1000 ED visits [5,10,12,34-37], and the ratio of females to males involved also varies [4,5,10,12,35,36,38,39]. In our study the frequency of poisoning-related ED visits was 4.2 per 1000 (around the middle of the published range) and females dominated, as has been found in some other countries [10,36,38,40]. Consistent with a recent study in southern Taiwan [20] and studies from other countries [6,8,10,35,38,40], attempted suicide was the most common (66.1%) cause of poisoning.

Drugs (49.9%) were the dominant poisoning agent in our study. This phenomenon has been reported in other regions of Taiwan [20], in many Western countries [1,4,6,37], and in some eastern Mediterranean [3] and Asian [38,39] countries. The implementation of the national health insurance program resulted in the wide availability of prescription drugs to the residents in Taiwan, which should be one of the major factors leading to the phenomenon observed in our study. Other contributing factors might include the prescription patterns of physicians, the dissemination of news related to suicides through the mass media, and user's knowledge about the effects of drug overdose.

We also found a negative association between exposure to hypnotics/sedatives and poisoning fatality. Most hypnotics/sedatives are considered to be relatively safe, and no case in our study involved an individual taking hypnotics/sedatives and pesticides (which caused most of the fatalities; 73.0%) at the same time. We believe these are the main reasons why exposure to drugs appears to be a protective factor in our study.

### Characteristics of fatality cases

In our study the poisoning-related fatality rate was 4.3%, which is very similar to that reported for Taiwan (5.7%) by the PCC [2]. This is much higher than in most Western countries [1,4,6,41], probably because of the wide use of highly toxic agrochemicals in Taiwan. Although drugs accounted for most cases of poisoning in our study, pesticides were a major contributor and paraquat exposure was a strong predictor of fatality. Pesticide exposure has also been reported to be a major poisoning problem in Taiwan [2,19,20] and some other countries [13,39,42,43]. Paraquat (24% w/v) is one of the most commonly used herbicides in Taiwan and has been the most common lethal agent of poisoning for a long time [2,24,44,45]. There are various explanations for the relatively large number of pesticide poisonings in Tainan. In particular, Tainan is located in an agricultural area, making pesticides easily accessible, and appropriate storage containers are not generally used. Another factor may be a lack of knowledge amongst the general public about the toxic effects of these chemicals. In addition to the measures that have been taken by authorities, including banning some of the most toxic pesticides (e.g., endosulfan and parathion) and promoting less use of pesticides, there is a need for greater efforts aimed at reducing the number of deaths caused by pesticide poisoning.

### Predictors of fatality

The diagnosis and treatment of acute poisoning are often challenging for ED staff, and some poisoning characteristics such as amount ingested [24], attempted suicide [28], pesticide exposure [28] and concomitant alcoholic consumption [46] have been found to be useful in risk stratification. However, the exposure history is sometimes hard to obtain from suicidal or critically ill patients, and information obtained at admission on illegal drug use is often incomplete or inaccurate [47]. In our study, information on the concomitant use of alcohol was only available in 62.4% of the enrolled patients. In the absence of accurate and adequate information on exposure, objective and obvious parameters for predicting fatality are valuable. Some scoring systems and biological predictors have been evaluated in previous studies for their application in risk stratification for certain herbicides. These include the Simplified Acute Physiology Score (SAPS) II [30,31], Acute Physiology and Chronic Health Evaluation (APACHE) II [26,30,31,33] and III [30,31] scores, blood pressure, FiO_2_, leukocytosis, acidosis, number of organ failures, pulmonary edema, and hyperkalemia [27,29,48]. Since accurate exposure history is often hard to acquire, simple and easy-to-obtain parameters that could be applied to most cases of poison exposure would be very useful in clinical settings. In this study, using multiple logistic regression analysis, we identified some easily measurable and useful parameters for predicting poisoning-related fatality, including body temperature, blood pressure, heart rate and respiration. Such objective information is routinely collected in the ED, and can help emergency physicians to quickly detect poisoned patients, predict poor outcomes, and expedite dispatch of those who need it to intensive care.

### Limitations of the study

As in other similar studies, the difficulty in obtaining complete information in some cases is a limitation of our study. For example, information on alcohol consumption was provided by only 62.4% of the participants, which limited further analyses of the related issues. It should also be noted that there are limitations in generally applying some of the results of this study to other regions. Even within the same area, the pattern of poisoning may vary over time, so regularly updating epidemiology data to identify trends for specific agents and to identify risk factors is important in developing prevention strategies.

## Conclusion

In this study we found that poisoning-related cases constituted only a small proportion (0.42%) of ED visits and had a relatively low fatality rate (4.2%). We also identified some simple parameters that can help risk stratification and detection of high risk patients, both of which are very important in provision of health care with the limited resources in the ED. While there may be limitations in generally applying the findings of this study to other regions, our results suggest that long-recognized indicators of a poor prognosis – old age and abnormal vital signs – can also be applied to cases of poisoning. In addition, we found that suicidal intent was a hallmark of poisoning-related fatality. Equally importantly, we demonstrated that even though the toxicity of paraquat is well recognized, it continues to stand out as the dominant agent for poisoning-related fatalities in some parts of the world. The results of this study should act as a reminder to clinicians, public health professionals and the general public of the serious consequences of exposures to this agent.

As described above, the patterns and causal agents of poisoning vary from region to region and with time in the same region. Regularly updating epidemiology data to detect trends for specific agents and to identify risk factors is necessary to enable public health practitioners to construct preventive strategies and assist clinicians in treating patients. Our study has provided an example of how such data can be collected.

## Competing interests

The author(s) declare that they have no competing interests.

## Authors' contributions

HLL initiated the study, designed the report form, recruited cases in one of the participating medical centers, performed data analyses and produced the first draft version of the paper. HJL and CHC supervised data collection in each of the participating medical centers, respectively, and helped design the study. Steve TYY recruited cases in one of the medical centers and helped design the report form. HRG conceived and designed the study, coordinated the overall research activities, supervised data analyses and contributed significant input to revisions of the manuscript. All authors approved and agreed to the final manuscript.

## Pre-publication history

The pre-publication history for this paper can be accessed here:


